# A Spectrally Interrogated Polarimetric Optical Fiber Sensor for Current Measurement with Temperature Correction

**DOI:** 10.3390/s23239306

**Published:** 2023-11-21

**Authors:** Tinko Eftimov, Georgi Dyankov, Petar Kolev, Veselin Vladev

**Affiliations:** 1 Photonics Research Center, Université du Québec en Outaouais, Rue 101 St-Jean Bosco, Gatineau, QC J8X 3G5, Canada; 2 Central Laboratory of Applied Physics, Bulgarian Academy of Sciences, 61 Sanct Peterburg Blvd., 4000 Plovdiv, Bulgaria; gdyankov@iomt.bas.bg (G.D.); v.p.vladev@abv.bg (V.V.); 3 Institute of Optical Materials and Technologies “Acad. J. Malinowski” (IOMT), Bulgarian Academy of Sciences (BAS), 109 “Acad. G. Bonchev” Str., 1113 Sofia, Bulgaria; p.kolev@iomt.bas.bg; 4 Photonics Laboratory, Department of Mathematics, Physics and Information Technologies, Faculty of Economics, University of Food Technologies, 26 Maritsa Blvd., 4000 Plovdiv, Bulgaria

**Keywords:** magnetic field sensor, current sensor, temperature independent measurements, polarimetry, fiber optic sensors, spectral interrogation

## Abstract

We report on a study of the temperature dependence of the response of a BSO crystal based polarimetric current sensor with spectral interrogation. Two possible interrogation schemes are discussed. The spectral dependence of the optical rotation along the crystal caused by temperature and current changes is investigated, and approximate dependences for the sensitivities to current *S*_I_ and temperature *S*_T_ are derived. A mixed term in the response with spectral interrogation is revealed, the elimination of which is achieved by tracking wavelength shifts Δλ_1_ and Δλ_2_ of two distinct extrema in the polarimetric response. A temperature independent second degree equation for the current changes Δ*I* as a function of the measured spectral shifts is derived and tested.

## 1. Introduction

Electric current and/or magnetic field fiber optic sensors have been known, developed and commercialized [1] for quite some time for the purposes of the power industry [2] and special applications [3]. These sensors are based either on the Faraday effect in the fiber itself, or in bulk materials [4] which use optical fiber transducers, such as fiber Bragg gratings (FBG), interferometers, or refractometers [5,6,7,8,9,10,11] in combination with magnetic field sensitive materials. These sensors are characterized by specific advantages and disadvantages as well as varying simplicity and complexity.

Among the simplest and most straightforward solutions are the polarimetric sensors using magneto-optical materials such as BSO (Bi_12_SiO_20_) and BGO (Bi_4_Ge_3_O_12_) crystals because of their high Verdet constant and because their wavelength dependence has been accurately measured and is well known [12,13]. The first polarimetric fiber optic sensor using BSO/BGO crystals [14,15], however, operated at a single wavelength and detected amplitude changes of the polarimetric response. These crystals are also temperature dependent [16,17], which limits the performance of the sensor. To account for temperature dependence, detection at two wavelengths has been proposed [18], or the temperature dependence of the BGO crystal has been taken into account [19]. The problem of the temperature dependence for the other types of current sensor has been addressed in different ways depending on the interrogation scheme and the magneto-sensitive material, using either temperature compensation [18,19,20,21,22,23] or simultaneous magnetic field and temperature measurement in the case of magnetic field sensors with spectral interrogation [24,25,26,27,28,29].

We have recently shown [30,31] that the well-known fluorescence in BSO crystals [32] can be used to generate a broad spectrum under ultraviolet or white LED excitation, which serves as a broadband source for a polarimetric scheme with spectral interrogation. We have also reported [33] that the BSO crystal based polarimetric sensor exhibits a wavelength dependent temperature response.

In the present work, we measure the optical activity of a BSO crystal at different temperatures, currents, and wavelengths and establish the spectral and temperature dependence of the intrinsic and the magnetic field induced polarization rotation. We show that, using a white LED, the spectrally interrogated polarimetric fiber optic current sensor can be used to eliminate the temperature dependence in the measurement of the electric current.

Section 2 presents the general experimental setup, the principle of operation, and the two possible interrogation techniques. Section 3 is devoted to the measurement of the spectral dependence of temperature-influenced optical activity from which we derive the spectral, temperature, and current dependences of the sensitivities to current and to temperature. To eliminate the temperature dependence, we use the spectral shifts of two extrema of the polarimetric spectral response and derive an expression for the current changes. The performance of the current sensor is simulated numerically.

## 2. Principle of Operation and Experimental Arrangement

### 2.1. Experimental Setup

**Figure 1:**
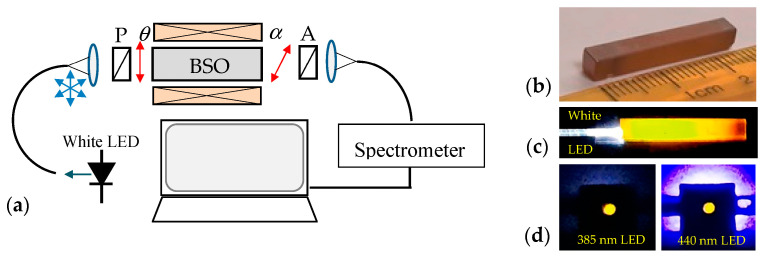
Polarimetric current sensor setup: (**a**) experimental arrangement, (**b**) side view of the BSO crystal used with dimensions, (**c**) top view of fluorescence and scattering with white LED illumination and (**d**) front views of fluorescence with 385 nm and 440 nm illumination via a large core optical fiber.

The experimental setup in Figure 1a was used to generate a broad spectrum from 520 nm to 800 nm in the BSO crystal and to observe the spectral distribution of the polarimetric responses. The side view with the crystal size are shown in Figure 1b, while Figure 1c shows the side glow caused by fluorescence and scattering from the crystal with white LED excitation, whose built-in blue LED is the major cause of fluorescence in the crystal. Figure 1d shows the front view of the fluorescent light coming out from the crystal when illuminated via a 1 mm diameter optical fiber with LEDs at 385 nm and 440 nm. A low cost film polarizer LPVISE2 × 2 (Thorlabs, Newton, NJ, USA) with extinction ratios of >100:1 for 400–740 nm, >1000:1 for 500–700 nm, and >5000:1 for 530–690 nm ranges was used in the experiments. The polarimetric response of the broadband spectrum is given by the expression [31]:

(1) $$I=\frac{1}{4}\left\{1+\cos\left[2\left(\phi +\theta -\alpha \right)\right]\right\}$$

In (1) *θ* and *α* are the polarizer and analyzer orientations and

(2) ϕ=ΔβL

is the accumulated phase along the circularly birefringent BSO crystal whose length is *L*, and Δ*β* = *β*_L_ – *β*_R_ is the propagation constant difference between the left and the right circularly polarized waves along the crystal. This difference is both wavelength and temperature dependent, and is expressed as [31]:

(3) $$\Delta \beta (\lambda ,T)=\rho (\lambda ,T)+V_{B}(\lambda ,T)B$$

**Figure 2:**
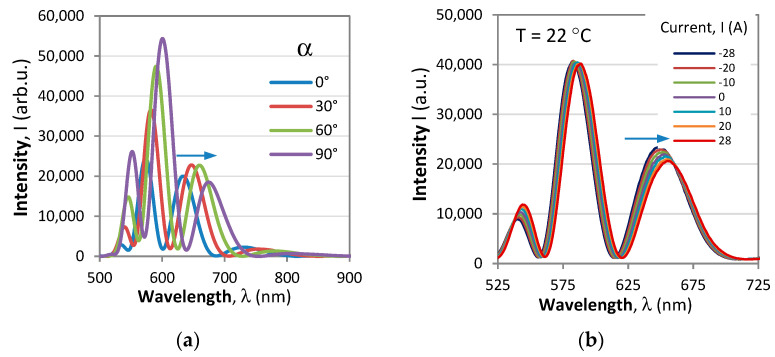
Spectral response of the polarimetric sensor: (**a**) as analyzer angle increases from 0° to 90°; (**b**) as current in the coil increases from −28 A to +28 A.

In (3) *ρ* (deg/mm) is the optical rotatory power of the crystal and the additional rotatory power *ρ*_F_ = *V*_B_*B* caused by the Faraday effect, *B* is the magnetic field and *V*_B_ is the Verdet constant. The wavelength dependences of *ρ*(*λ*) and *V*_B_(*λ*) are well known [12,13], both decrease with wavelength and *V*_B_ is proportional to *ρ* [17]. However, the temperature dependences have been insufficiently studied and later in the paper we present more detailed results on them. Inserting (3) and (2) into (1), we have, for the wavelength and temperature dependent intensity *I*(*λ*, *T*) [30,31]:

(4) $$I(\lambda ,T)=\frac{1}{4}\left\{1+\cos\left[\Phi (B,T,\lambda )\right]\right\}=\frac{1}{4}\left\{1+\cos\left[2\left(\rho (\lambda ,T)+V_{B}(\lambda ,T)B\right)L+2\left(\theta -\alpha \right)\right]\right\}$$

with Φ(*B*, *T*, *λ*) being the total phase in the polarimetric response. The typical spectral responses of a polarimetric sensor to changes of the polarizer/analyzer orientations and to current (magnetic field) are shown in Figure 2. The plots show that the particular position of the modulated pattern can be fine-tuned by changing the orientation of the analyzer/polarizer via the *θ* − *α* term. The changes of the measurand (current/magnetic field) and of the temperature lead to shifts of the polarimetric response, as shown in Figure 2b.

### 2.2. Principle of Operation and Sensitivities

Unlike the single wavelength polarimetric scheme [14] in which the intensity (4) is measured, in our arrangement, we observe by a spectrometer the whole spectrum, which exhibits an oscillatory response. Since both *ρ*(*λ*) and *V*_B_(*λ*) decrease with wavelength, the period of the wavelength dependent response Λ (which is the free spectral range–FSR) increases with wavelength. As the angle between polarizer and analyzer can be varied, we can tune the phase 2(*θ* − *α*) and thus fix the position of the polarimetric response.

### 2.3. Interrogation Detection Techniques

There are two detection techniques that can be used to detect the changes in the polarimetric spectral response. In the first we track the wavelength position of a minimum *λ*_m_ or a maximum *λ*_M_, as the magnetic field/electric current changes. Thus, the wavelength shifts δλ_M_ = δλ_M_ − δλ_M,0_ of a maximum and δλ_m_ = δλ_m_ − δλ_m,0_ of a minimum, defined with respect to some wavelength positions at *I* = 0 A and *T* = *T*_0,_ are tracked. In the second, we observe the intensities that are +π/2 and –π/2 phase-shifted with respect to an extremum, namely *I*_M_^+^ and *I*_M_^−^ for a maximum or *I*_m_^+^ and *I*_m_^−^ for a minimum, and calculate the differences and the sums of intensities that are π-shifts with respect to one another: Δ_M_ = *I*_M_^+^–*I*_M_^−^, Σ_M_ = *I*_M_^+^ + *I*_M_^−^ and Δ_m_ = *I*_m_^+^ − *I*_m_^−^, Σ_m_ = *I*_m_^+^ + *I*_m_^−^ and then calculate the normalized differences *N*_M_ = Δ_M_/Σ_M_ and *N*_m_ = Δ_m_/Σ_m_ which change with current/magnetic field and with temperature.

#### 2.3.1. Extrema Wavelength Shifts

In this case, for a given magnetic field *B* (or current *I*) and temperature *T*, the wavelengths *λ*_M,k_ and *λ*_m,k_ of the *k*-th maximum and minimum of the polarimetric response are those for which the phase Φ from (4) is:

(5a) $$\Phi_{M}(B,T,\lambda_{M,k})=2k\pi \;\max\;\mathrm{and}\;\Phi_{m}(B,T,\lambda_{m,k})=(2k+1)\pi \;\min$$

and the phase change ΔΦ is then

(5b) $$\Delta \Phi_{k}=\frac{\partial \Phi_{k}}{\partial \lambda }\Delta \lambda_{k}+\frac{\partial \Phi_{k}}{\partial I}\Delta I+\frac{\partial \Phi_{k}}{\partial T}\Delta T$$

As mentioned in [30,31], the periodic response is caused by the spectral dependence of the optical activity *ρ*(λ) and the Verdet constant *V*_B_(λ). When we track the shifts Δλ_k_ of an extremum (at *λ*_M,k_ or *λ*_m,k_) with respect to the position for *I* = 0 (*B* = 0) caused by changes of the magnetic field (or current) Δ*B* (or Δ*I*) and of the temperature Δ*T*, the resultant phase change ΔΦ of the phases Φ_M_ or Φ_m_ is zero because the condition (5a) for the tracked extremum remains constant i.e., Φ_M_ = *2kπ* = *const* and Φ_M_ = *2kπ* = *const*. The expression (5b) becomes null, and solving with respect to Δλ we obtain:

(6a) $$\Delta \lambda_{k}=\lambda_{k}-\lambda_{k,0}=-\left(\frac{\partial \Phi }{\partial B}/\frac{\partial \Phi }{\partial \lambda }\right)\Delta B-\left(\frac{\partial \Phi }{\partial T}/\frac{\partial \Phi }{\partial \lambda }\right)\Delta T=S_{B}\Delta B+S_{T}\Delta T$$

where *S*_B_ and *S*_T_ are the sensitivities to magnetic field and temperature changes:

(6b) $$S_{B}=\frac{\Delta \lambda_{k}}{\Delta B},\;S_{T}=\frac{\Delta \lambda_{k}}{\Delta T}$$

**Figure 3:**
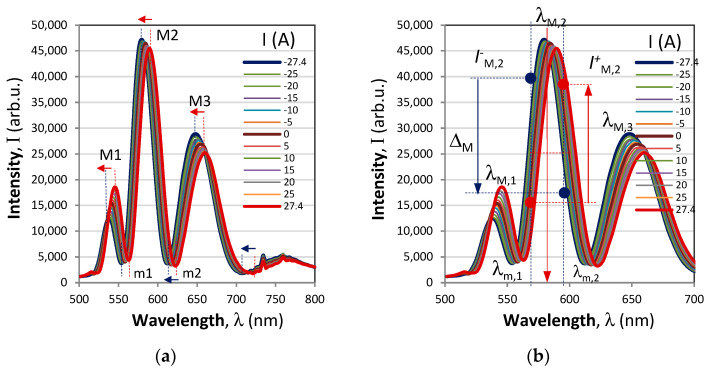
Interrogation techniques: (**a**) extrema wavelength shift; (**b**) π−shifted differential shift.

**Figure 4:**
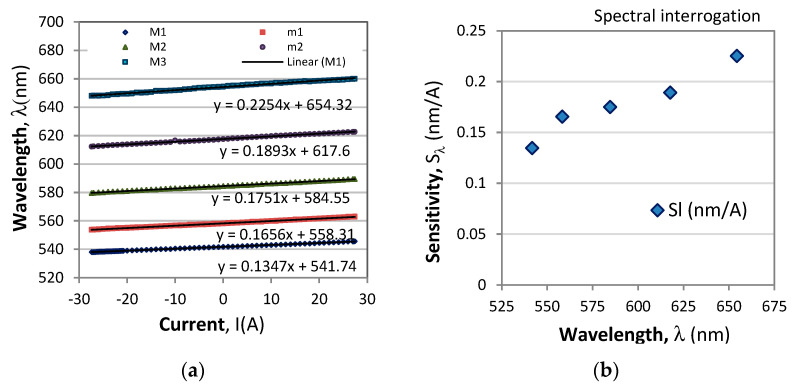
Spectral responses of the extrema shift interrogation technique: (**a**) wavelength shifts of the maxima M_1_, M_2_, M_3_ and the minima m_1_ and m_2_, (**b**) wavelength dependence of the sensitivities *S*_I_(λ).

In (5a,b) and (6a,b) the instantly tracked *λ*_k_ is either *λ*_M,k_ or *λ*_m,k_ and Δ*λ*_k_ is the wavelength shift of the extremum from the initial value *λ*_k,0_ for which *I* = 0 and *T* = *T*_0_. This type of interrogation is illustrated in Figure 3a. Both the magnitude and the sign of the current/magnetic field can thus be measured. Figure 4a shows the wavelength shifts of the extrema from the response in Figure 3a, which prove to be linear with wavelength, while Figure 4b shows the correspondingly measured sensitivities which increase with wavelength [30,31] because they are inversely proportional to dispersion of the optical activity and the Verdet constant.

#### 2.3.2. π-Shifted Normalized Differential Response

In this case, the intensities $I_{k}^{¨+}$ and $I_{k}^{¨-}$ of the polarimetric response around the *k*-th extremum ($I_{M,k}^{¨\pm }$ and $I_{m,k}^{¨\pm }$) that are π-shifted to each other can be defined as:

(7) $$I_{k}^{¨+}=I_{0}\cos\left[2\varphi_{k}+2(\theta -\alpha )+\frac{\pi }{2}\right]I_{k}^{¨-}=I_{0}\cos\left[2\varphi_{k}+2(\theta -\alpha )-\frac{\pi }{2}\right]$$

The normalized differential responses around the *k*-th maximum or the minimum are defined as:

(8) $$N_{M,k}=\frac{I_{M,k}^{¨+}-I_{M,k}^{¨-}}{I_{M,k}^{¨+}+I_{M,k}^{¨-}},\;N_{m,k}=\frac{I_{m,k}^{¨+}-I_{m,k}^{¨-}}{I_{m,k}^{¨+}+I_{m,k}^{¨-}}$$

In the initial state $I_{M,k}^{¨+}$ = $I_{m,k}^{¨-}$, but when the polarimetric response shifts to the right the difference $\Delta =I_{M,k}^{¨+}-I_{M,k}^{¨-}$ becomes positive and vice versa, as illustrated in Figure 3b by the red and blue vertical arrows. Therefore, both the amplitude and the sign of the current/magnetic field can be detected. The sensitivities to magnetic field/current and to temperature are defined in this case as:

(9) $$\Pi_{B,k}=100\frac{\Delta N_{k}}{\Delta B}\;\mathrm{or}\;\Pi_{I,k}=100\frac{\Delta N_{k}}{\Delta I},\;\Pi_{T,k}=100\frac{\Delta N_{k}}{\Delta T}$$

**Figure 5:**
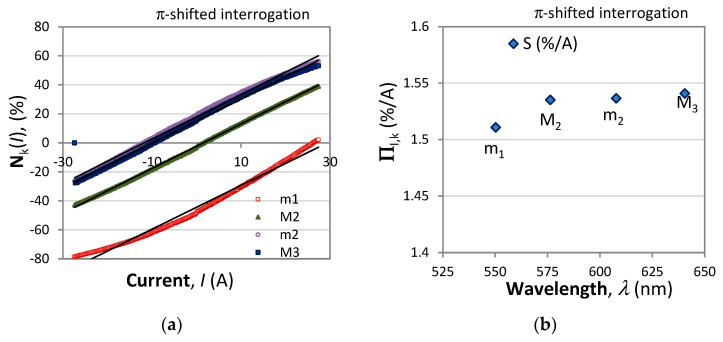
Normalized differential responses of the π-shifted interrogation technique: (**a**) responses of the maxima M_1_, M_2_, M_3_ and the minima m_1_ and m_2_, (**b**) wavelength dependence of the sensitivities П_I_(λ) around the maxima M_2_, M_3_ and the minima m_1_ and m_2_.

Figure 5 presents the responses of the normalized differential signals according to the π-shifted interrogation technique. As is seen, the responses have different sensitivities, though not quite as linear as those from Figure 4. The sensitivity in this case shows similar wavelength dependence as with the first interrogation method (Figure 4b).

#### 2.3.3. Comparison

In the present paper we present a more detailed analysis of a current sensor with temperature correction using the first scheme; however, some general comparisons between the two interrogation schemes can be outlined as follows:

(i) The responses of the π-shifted method exhibit nonlinearities compared with the extrema shift method;
(ii) The normalized differential signal is faster to calculate compared with the extrema shift method and is more appropriate if fast changing short circuit current changes are to be detected, in which case slowly varying temperature induced noise is irrelevant;
(iii) The resolution of the π-shifted method is better because the signals are away from minima and less sensitive to noise.

## 3. Experiment and Results

### 3.1. Experimental Setup

**Figure 6:**
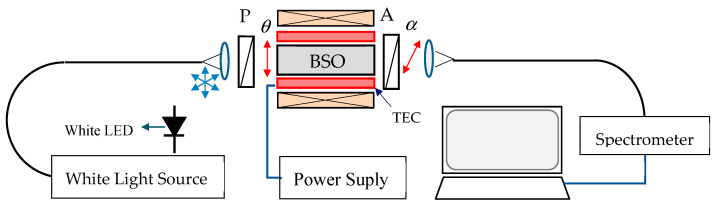
Experimental setup.

To study the possibility for temperature corrected current measurements, we need to know the dependences of the optical activity and the Verdet constant on the wavelength *ρ*(λ), *V*(λ) and on temperature *ρ*(*T*), *V*(*T*). The experimental setup used to obtain the needed measurements is shown in Figure 6.

The light source was a white halogen lamp (Ocean Optics) and the spectrometer was an Avantes VASPEC-ULS2048CL-EVO, (0.5 nm resolution and a 400 nm to 900 nm range). The light from the source was coupled to a 600 μm large core quartz-polymer lead-in fiber, collimated at its output, polarized by a polarizer through an angle *θ*, traversing the BSO crystal, passed through an analyzer oriented to an angle *α*, and focused to the same type of a lead-out fiber. Alternatively, for current and temperature measurements, the white light halogen lamp was replaced by a white LED. The BSO crystal (4 × 4 × 25) was placed in an aluminum holder and was heated/cooled using a thermoelectroc cooler (TEC). The polarizer was fixed with the transmission axis along the horizontal axis of the crystal. Only the analyzer was rotated during measurements of the optical activity and its temperature dependence. The temperature could be varied from −32 °C to 62 °C. The power supply could provide a current in the range from −30 A to +30 A (±0.1 A) which fed the solenoid coil to create a homogeneous magnetic field along the BSO crystal, as shown in Figure 6.

### 3.2. Results of Optical Activity Measurements

Using the setup shown in Figure 6, we performed a sequence of measurements in which we changed the temperature *T* and current *I* across the solenoid and measured the output angle of rotation of the polarization at a particular wavelength λ. The measurements were made by compensation of the polarization rotation via turning the analyzer angle *φ* so that a given minimum at a specified wavelength in the polarimetric response (Figure 2) remained at the same position, which meant that for a given minimum (*k* = constant) as

(10) $$\Phi_{k}=2\left[\rho (\lambda_{k},T)+V_{I}(\lambda_{k},T)I\right]L+2\left(\theta -\varphi \right)=(2k+1)\pi$$

The sequence of measurements was as follows:

(i) A wavelength from among the following 540 nm, 570 nm, 600 nm, 630 nm, 660 nm and 690 nm was chosen, starting from the highest value. In the absence of magnetic field (*I* = 0 A) and at room temperature (≈22 °C) the analyzer was rotated until a minimum of the response coincided with the chosen wavelength of measurement (690 nm for example);
(ii) For a chosen value of the wavelength λ_k_, a value of the current as fixed between *I* = −30 A and *I* = 30 A. For a fixed value of the current, the temperature was varied between *T* = −30 °C and 60 °C, and for each temperature the analyzer was rotated to compensate for the temperature and current induced phase changes (26a,b) due to the *ρ*(λ_k_,*T*), *V_I_*(λ_k_,*T*) and *I,* and the particular value for *φ* was found;
(iii) After the temperature was scanned, a new value for the current for the same wavelength was set and then the temperature scanning was repeated;
(iv) After all currents were scanned, the next wavelength was chosen and the procedure from (i) to (iii) was repeated.

The results obtained provided the possibility for the following analysis to be performed.

First, the only spectral and temperature dependence was in the optical activity and the Verdet constant, and second, neither of them depended on the current, so the compensation angle can be represented as.

(11) $$\varphi (\lambda ,I,T)=V_{I}(\lambda ,T)L.I+\rho (\lambda ,T)L\;\mathrm{with}\;V_{I}(\lambda ,T)I=V(\lambda ,T)B$$

Second, we analyzed the results for *I* = 0, which revealed the dependences *ρ*(λ_k_, *T*), so we have

(12) $$\varphi_{0}(\lambda ,T)=\varphi (\lambda ,0,T)=\rho (\lambda ,T)L$$

**Figure 7:**
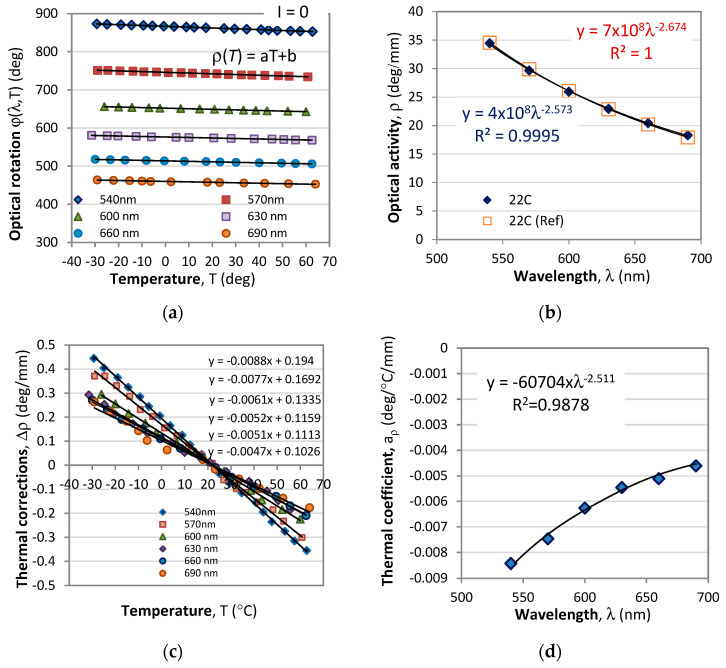
Temperature and spectral dependences of the optical rotation without magnetic field (*I* = 0): (**a**) rotation angle vs. temperature at different wavelengths; (**b**) wavelength dependence of the optical activity *ρ*(λ) at room temperature (22 °C); (**c**) temperature corrections Δ*ρ* to the optical activity with respect to the response from (**b**) at room temperature; (**d**) thermal coefficient *a_ρ_*.

Figure 5a shows a plot of the rotation angle caused by optical activity vs. temperature for the above six wavelengths in the absence of a magnetic field (*I* = 0). As is seen from this figure, the optical rotation linearly reduces with temperature and can be represented in the form:

(13) $$\varphi_{0}(\lambda ,T)=\rho (\lambda ,T)L=\left\lfloor a_{\rho }(\lambda )T+\rho_{0}(\lambda )\right\rfloor L$$

where the thermal coefficient *a_ρ_*(λ) that determines the slope of the lines in Figure 7a as well as the optical activity *ρ*_0_ are wavelength dependent.

**Figure 8:**
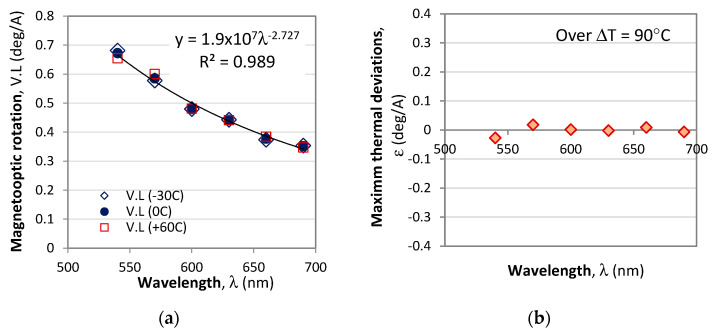
Spectral and temperature dependence of the magneto-optical rotation without: (**a**) *V*(λ)*L*; (**b**) the maximum thermally induced variations over a 90 °C temperature range.

Third, from the measurements at different temperatures in the −30 °C to 60 °C range for different currents from −25 A to +25 A, we can retrieve the optical rotation *V*_I_.*L* (deg/A) due to the magneto-optic effect and its wavelength dependence presented in Figure 8. In that figure we plot *V*_I_(λ). *L* for three temperatures, −30 °C, 0 °C, and 60 °C, and it is seen that the temperature deviations vary randomly in either direction. The relative error due to temperature-dependent deviations of the magneto-optic activity varied with wavelength, and, on average, was found to be ≈0.7%. 

We thus conclude that

(14) $$V_{I}(\lambda ,T)=V(\lambda )+\varepsilon (T)\approx V(\lambda )$$

where *ε*(*T*) is a negligible thermally dependent correction.

### 3.3. Approximations 

The experimental plots obtained for *ρ*_0_(λ), *a_ρ_*(λ) and *V*(λ) are found to be sufficiently well approximated by power law functions, namely as:

(15a) $$\rho_{0}(\lambda )=R_{0}\lambda^{-r}\;R_{0}=4\;\times \;10^{8},\;r=-2.573\;(R^{2}=0.9995)$$

(15b) $$a_{\rho }(\lambda )=A_{0}\lambda^{-a}\;A_{0}=-60704,\;a=-2.511\;(R^{2}=0.9878)$$

(15c) $$V_{I}(\lambda )=V_{0}\lambda^{-v}\;V_{0}=760533,\;v=-2.728\;(R^{2}=0.9896)$$

Based on the above we can write (14) as

(16a) $$\varphi (\lambda ,I,T)=\rho (\lambda ,T)L+V_{I}(\lambda )L.I=a_{\rho }(\lambda )T.L+\rho_{0}(\lambda ).L+V_{I}(\lambda )I.L$$

and for λ from 540 nm to 700 nm and *T* from −30 °C to +60 °C, the power law approximations (21) can be used for all wavelength dependences:

(16b) $$\varphi (\lambda ,I,T)=\left\lfloor A_{0}\lambda^{-a}T+R_{0}\lambda^{-r}+V_{0}\lambda^{-v}.I\right\rfloor .L$$

## 4. Simultaneous Current and Temperature Measurement Technique

### 4.1. Sensitivities to Current and Temperature

To realize temperature corrected current measurement the non-linear power-law approximations for *a_ρ_*(λ), *ρ*_0_(λ) and *V*(*λ*) are simplified according to the following procedure:

(i) Use the power law approximations (21) to study the responses to current and temperature changes and determine the sensitivities *S*_I_ and *S*_T_;
(ii) Study the wavelength, temperature, and current (magnetic field) dependences of *S*_I_ and *S*_T_;
(iii) Develop a method for simultaneous two-parameter measurement.

We first model the white light LED spectral distribution used in the sensor by a shifted Gamma function defined as [31]:

(17) $$S(\lambda )=\frac{\beta^{\alpha }}{\Gamma (\alpha )}{\left(\lambda -\lambda_{0}\right)}^{-\alpha }e^{-\beta \left(\lambda -\lambda_{0}\right)}$$

Taking into account (21) and (22a,b), the intensity distribution at the analyzer can be represented as

(18) $$I(\lambda ,I,T)=\frac{I_{0}}{4}S(\lambda )\left\{1+\cos\left[\left(A_{0}\lambda^{-a}T+R_{0}\lambda^{-r}+V_{0}\lambda^{-v}.I\right)L+2\left(\theta -\varphi \right)\right]\right\}$$

The parameters for the Gamma function are as follows: λ_0_ = 515 nm, α = 3.75 and *β* = 27. The theoretical fit using (17) a real polarimetric response at analyzer angle 0° is presented in Figure 7 and is compared with the response at 60°.

The full differential of the phase per unit length dΦ/2L is found from (3b)

(19a) $$\frac{d\Phi }{2L}=\left(-aA_{0}\lambda^{-a-1}T-rR_{0}\lambda^{-r-1}-vV_{0}\lambda^{-v-1}.I\right)d\lambda +\left(-vV_{0}\lambda^{-v-1}\right)dI+\left(A_{0}\lambda^{-a}\right)dT$$

(19b) $$\frac{d\Phi }{2L}=F_{\lambda }d\lambda +F_{I}dI+F_{T}dT$$

In case of spectral interrogation by extremum tracking, *d*Φ = 0, and for *δλ* we find:

(20a) $$d\lambda =-\frac{F_{I}}{F_{\gamma }}dI-\frac{F_{T}}{F_{\lambda }}dT=S_{I}dI+S_{T}dT$$

(20b) $$S_{I}=\frac{d\lambda }{dI}=-\frac{F_{I}}{F_{\gamma }},\;S_{T}=\frac{d\lambda }{dT}=-\frac{F_{T}}{F_{\gamma }}$$

**Figure 9:**
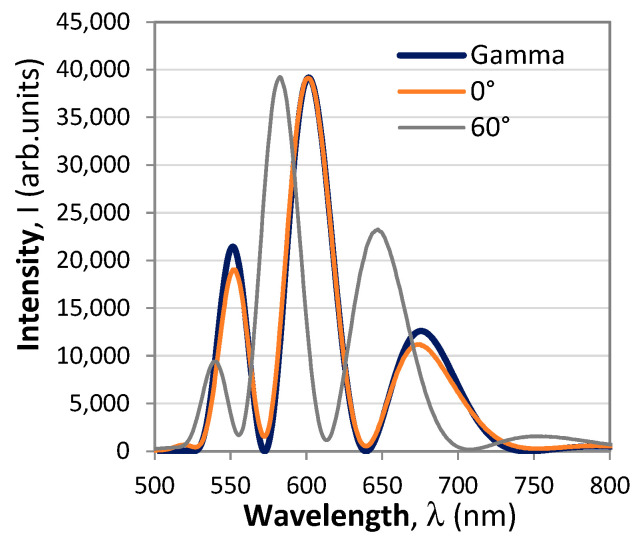
Two responses of the polarimetric sensor for the analyzer turned at 0° and 90° given by the thin line. The thick line is a theoretical fitting to the response at 0° from (18).

As the power law expressions are non-linear, we proceed to the second step outlined above and perform a study of the wavelength, temperature, and current (magnetic field) dependences of *S*_I_(*λ*, *I*, *T*) and *S*_T_(*λ*, *I*, *T*). To do that, we change the current *I* at a constant temperature *T*, and then change temperature for constant current and measure the resulting wavelength shifts of the minima and maxima in the distribution from Figure 9. Then, we plot the dependence of Δ*λ* vs. *I* for different temperatures for each extremum, as well as Δ*λ* vs. *T* for different currents, from which we determine the needed sensitivities.

(21) $$\Delta \lambda =S_{I}(\lambda ,T)\Delta I+S_{T}(\lambda ,I)\Delta T$$

**Figure 10:**
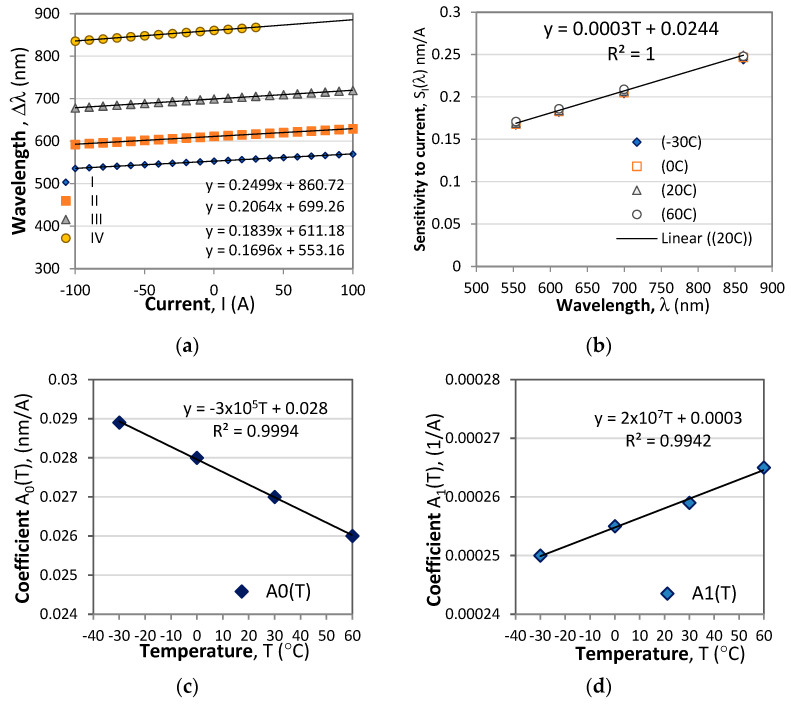
Responses to current changes: (**a**) shifts of the extremum wavelength vs. current changes from −100 A to 100 A; (**b**) wavelength dependence of the sensitivity to current *S*_I_(λ); (**c**) the temperature dependence of the coefficient *A*_0_(T) from (22a); (**d**) the temperature coefficient *A*_1_(*T*).

Figure 10 shows the wavelength changes of four minima. The wavelength dependence for the current sensitivity *S*_I_(λ) is experimentally confirmed in ref. [31], and, as we see at each wavelength, the Δ*λ*(*I*) dependence is linear. So is the *S*_I_(λ) over the range of wavelengths above 550 nm, though weakly dependent on temperature. As is seen from Figure 10b, the sensitivity to current is linearly dependent on the wavelength and can be represented as follows:

(22a) $$S_{I}(\lambda )=A_{0}(T)+A_{1}(T)\lambda \;(nm/A)$$

The linear fits of each of the coefficients presented in Figure 10c,d below can be represented as follows:

(22b) $$A_{0}(T)=A_{00}+A_{01}T\;(\mathrm{nm}/A)\;\mathrm{and}\;A_{1}(T)=A_{10}+A_{11}T\;(1/A)$$

**Figure 11:**
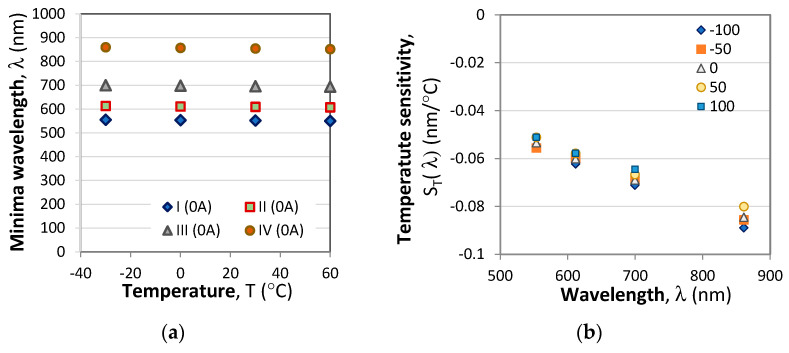

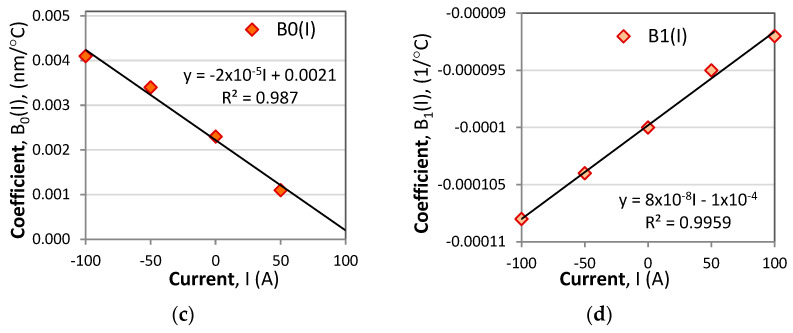
Temperature dependence: (**a**) Temperature induced wavelength shifts of four minima at I = 0A; (**b**) Wavelength dependence of sensitivities to temperature *S*_T_(λ) for different currents; (**c**) temperature dependence of the B0 coefficient from (23a); (**d**) the thermal coefficient B_1_from (23).

The wavelength shifts of the four minima with temperature and the temperature sensitivity on the wavelength *S*_T_(λ) at different current levels are presented in Figure 11.

From Figure 9b it is seen that the temperature sensitivity *S*_T_(λ*,I*) is linearly dependent on the wavelength and can be written as:

(23a) $$S_{T}(\lambda ,I)=B_{0}(I)+B_{1}(I)\lambda \;(nm/{}^{\circ}C)$$

where *B*_0_(*I*) and B_1_(*I*) are also linear functions of the current Figure 9c,d and are written as:

(23b) $$B_{0}(I)=B_{00}+B_{01}I\;(\mathrm{nm}/{}^{\circ}C)\;\mathrm{and}\;B_{1}(T)=B_{10}+B_{11}I\;(1/{}^{\circ}C)$$

As follows from (21)

$$\lambda -\lambda_{0}=S_{I}(\lambda ,T)\left(I-I_{0}\right)+S_{T}(\lambda ,I)\left(T-T_{0}\right)$$

### 4.2. Two Points Method for Simultaneous Two-Parameter Measurement

In this form, the unknown quantities are the current *I* and temperature *T* and after insertion of (22b) into (22a) and of (23b) into (23a) and, after some rework for the *k*-th extremum tracked, we obtain

(24) $$\lambda_{k}-\lambda_{k0}=\Delta \lambda_{k}=A_{k}\Delta I+B_{k}\Delta T+C_{k}\Delta I\Delta T$$

where

(25a) $$A_{k}=A_{00}+A_{10}\lambda_{k}-A\prime_{k}T_{0}\;A\prime_{k}=A_{01}+A_{11}\lambda_{k}$$

(25b) $$B_{k}=B_{00}+B_{10}\lambda_{k}-B\prime_{k}I_{0}\;B\prime_{k}=B_{01}+B_{11}\lambda_{k}$$

(25c) $$C_{k}=A\prime_{k}+B\prime_{k}$$

Equation (24) contains a mixed term proportional to Δ*I*Δ*T*. To eliminate the temperature dependence, we chose two extrema wavelengths λ_1_ and λ_2_ (*k* = 1, 2) to track to our choice. By varying the orientation of the analyzer we can fine tune the position of the pattern. Equation (24) then is written for each of the wavelengths as:

(26a) $$\Delta \lambda_{1}=A_{1}\Delta I+B_{1}\Delta T+C_{1}\Delta I\Delta T$$

(26b) $$\Delta \lambda_{2}=A_{2}\Delta I+B_{2}\Delta T+C_{2}\Delta I\Delta T$$

Solving (26a,b) with respect to the mixed ΔT term and eliminating the temperature dependence leads to a quadratic equation with respect to the current:

(27) $$a\Delta I^{2}+b\Delta I+c=0$$

where

(28) $$a=A_{1}C_{2}-C_{1}A_{2},\;b=\Delta \lambda_{2}C_{1}-\Delta \lambda_{1}C_{2}+A_{1}B_{2}-B_{1}A_{2},\;c=\Delta \lambda_{2}B_{1}-\Delta \lambda_{1}B_{2}$$

Solving (27) with respect to Δ*I* yields

(29) $$\Delta I=\frac{-b-\sqrt{b^{2}-4ac}}{2a}$$

The electric current measurement procedure runs thus as follows:

(i) The coefficients from Table 1 are substituted into (25a–c);
(ii) By fixing the position of the analyzer with respect to the polarizer, a desirable position of the spectral response is fixed for *I*_0_ = 0 and *T* = *T*_0_ which quantities are also to be inserted into (25a–c). The two extrema whose shifts are to be monitored are fixed, and under these conditions their values λ_10_ and λ_20_ are measured and substituted into (25a–c) as well;
(iii) The instant values of the extrema λ_1_ and λ_2_ are measured over regular intervals *t* and are inserted into (25a–c) and into (28) for the coefficients *a*, *b*, and *c* from (27);
(iv) The current change is calculated (29).

To test the method, we perform the above procedure by setting in (29) the current *I* from −100 A to +100 A for temperatures *T* = −30 °C, 0 °C, 30 °C, 60 °C, and 90 °C which causes the spectral response to shift. For each combination of current and temperature we determine the values of the wavelengths λ_1_, λ_2,_ and λ_3_ of the three observable minima, which values are inserted into (25a–c). The reference wavelength values λ_1,0_ = 525.7 nm, λ_2,0_ = 572.8 nm, and λ_3,0_ = 639.7 nm from (24) are at *T*_0_ = 0 and *I*_0_ = 0. The pairs of wavelengths to determine the current are (λ_1_, λ_3_) and (λ_2_, λ_3_). Figure 9 shows the correspondence between the preset value of the current *I* and the calculated *I*_c_ for each of the pairs from Equation (29).

**Figure 12:**
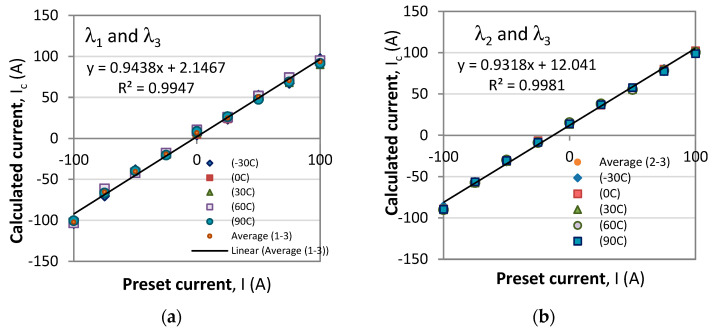
A linear fit for the orrespondence between calculated current and preset current: (**a**) for the (λ_1_, λ_3_) pair; (**b**) for the (λ_2_, λ_3_) pair.

**Figure 13:**
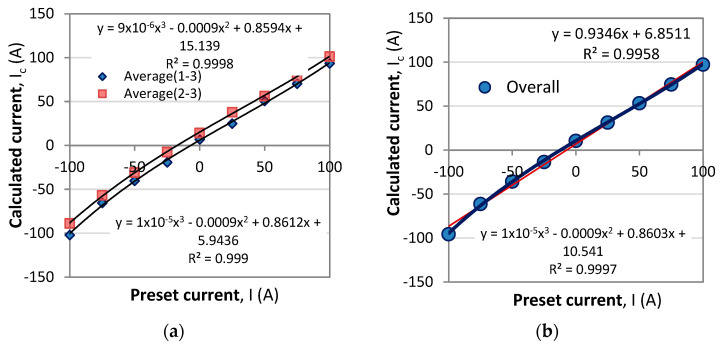
A third degree polynomial fit for the orrespondence between calculated current and preset current: (**a**) for the (λ_1_, λ_3_) pair; (**b**) for the (λ_2_, λ_3_) pair.

The results obtained for the temperatures from −30 °C to +90 °C reveal that the correspondence is linear and close to an identity function, yet from the linear fits, the proportionality coefficient is 0.9438 for the first pair and 0.9316 for the second (Figure 12a,b). We first note that, on average, the responses are the same for all temperatures. Second, a certain non-linearity is observed, and third, the two correspondences are differently offset from the origin of the coordinate system. Figure 13a shows the correspondences averaged over all temperatures with a third-order polynomial. Figure 13b shows the average of the two responses from Figure 13a. The first and third order polynomials are summarized in Table 2. Figure 12 and Figure 13 reveal that the slight non-linearity is observed at the extremities for negative currents. These we assume are due to the neglect of the weak temperature dependence of the Verdet constant from Figure 8b and to extending by a factor of more than three the current range in the simulations compared with the range of measurements to determine the sensitivities. The high values of the coefficient of determination *R*^2^ for the third-order polynomial mean that a convenient look-up table can be compiled to list the correct value of the preset current.

Having presented the principle of temperature correction using wavelength shifts at two different wavelengths, we can summarize the advantages of spectral interrogation as follows:

First, since sensitivities to current and temperature vary with wavelength, by measuring the responses at two different wavelengths it is possible to eliminate the temperature dependence.

Second, the normalized differential π-shifted response and the signals measured to track the spectral shifts do not depend on the power level of the source and are immune to power fluctuations.

Third, the optical scheme with a broadband source and spectral interrogation is simpler that those at two wavelengths with temperature compensation which require polarization beamsplitters, fiber optic splitters [18], or those based on magnetostrictive materials and magnetic fluids [20,21,22,23,24,25,26,27,28,29].

## 5. Conclusions

The performed measurements, subsequent analysis, and simulations of a polarimetric current sensor with spectral interrogation allow us to draw the following conclusions:

1. Two types of spectral interrogation technique could be used: Wavelength shifts of the minima and/or maxima and normalized differential intensity response at pairs of wavelengths which are *π*-shifted over the spectral range. In both cases the sensitivities to current are wavelength dependent;
2. We performed detailed measurements on the temperature, current, and spectral dependences of the intrinsic and magnetic field induced optical activity of BSO crystals in the range from 540 nm to 690 nm, current range from −30 to +30 A, and temperature range from −30 °C to 60 °C;
3. The temperature dependence of the intrinsic optical activity *ρ*(λ,T) was found to be linear in the form *ρ*(λ,T) = *ρ*_0_(λ) + *a*(λ)*T* within the above range, while the wavelength dependence of the coefficients *ρ*_0_(λ) and *a_ρ_*(λ) could be fitted with coefficients of determination of R^2^ = 0.9878 or better;
4. The temperature dependence of the Verdet constant was found to be very weak, and over a Δ*T* = 90 °C temperature range is less than 1.08 × 10^−3^ deg/A/mm, typically <7.2 × 10^−4^ deg/A/mm above 570 nm. The wavelength dependence of the Verdet constant could be fitted by a power law with R^2^ = 0.989;
5. On the basis of the above established approximations, it was found that the wavelength shift of an extremum is a linear combination of the current and temperature changes, but contains a mixed term. By making use of spectral shifts at two extrema λ_1_ and λ_2_, the temperature dependence was lifted and a third-order polynomial equation for current changes Δ*I* was derived;
6. A straightforward current measurement procedure was proposed and tested numerically.

## Figures and Tables

**Table 1:** Coefficients for the expressions for the sensitivities *S*_I_ and *S*_T_.

A_00_ (nm/A)	A_01_ (nm/°C/A)	A_10_ (1/A)	A_11_ (1/°C /A)	B_00_ (nm/°C)	B_01_ (nm/°C/A)	B_10_ (1/°C)	B_11_ (1/°C/A)
2.8 × 10^−2^	−3 × 10^−5^	2.548 × 10^−4^	2 × 10^−7^	2.1 × 10^−3^	−2.05 × 10^−5^	−9.97 × 10^−5^	8.2 × 10^−8^

**Table 2:** Coefficients of the linear and third-order polynomial fits for the correspondence plots of the calculated *I*_c_ versus preset value of the current *I*.

Pairs	Linear	Third-Order Polynomial
(λ_1_, λ_3_)	*I*_c_ = 0.9438 *I* + 2.1467 (R^2^ = 0.9947)	*I*_c_ = 1 × 10^−5^ *I*^3^ – 0.0009 *I*^2^ + 0.8612 + 5.9436 (R^2^ = 0.999)
(λ_2_, λ_3_)	*I*_c_ = 0.9318 *I* + 12.041 (R^2^ = 0.9981)	*I*_c_ = 9 × 10^−6^ *I*^3^ – 0.0009 *I*^2^ + 0.8594 + 15.139 (R^2^ = 0.9947)
Overall	*I*_c_ = 0.9346 *I* + 6.8511 (R ^2^= 0.9958)	*I*_c_ = 1 × 10^−5^ *I*^3^ – 0.0009 *I*^2^ + 0.8603 + 6.8511 (R^2^ = 0.9997)

## Data Availability

Data are contained within the article.
